# Reducing Heat Without Impacting Quality: Optimizing Trypsin Inhibitor Inactivation Process in Low-TI Soybean

**DOI:** 10.3390/foods14173039

**Published:** 2025-08-29

**Authors:** Ruoshi Xiao, Luciana Rosso, Troy Walker, Patrick Reilly, Bo Zhang, Haibo Huang

**Affiliations:** 1 Department of Food Science and Technology, Virginia Polytechnic Institute and State University, Blacksburg, VA 24060, USA; rosexiao@vt.edu (R.X.); troymw@vt.edu (T.W.); 2 School of Plant and Environmental Sciences, Virginia Polytechnic Institute and State University, Blacksburg, VA 24060, USA; luciana@vt.edu; 3 Department of Biological Systems Engineering, Virginia Polytechnic Institute and State University, Blacksburg, VA 24060, USA; pwpoinsettia@vt.edu

**Keywords:** soybean meal, trypsin inhibitor, protein digestibility, amino acids

## Abstract

A soybean meal is a key protein source in human foods and animal feed, yet its digestibility is constrained by endogenous trypsin inhibitors (TIs). Thermal processing is the mainstream tool for TI inactivation, but high-intensity heat treatments increase energy consumption and can potentially denature proteins, diminishing nutritional quality. Reducing the thermal input while maintaining nutritional quality is, therefore, a critical challenge. One promising strategy is the use of soybean cultivars bred for low-TI expression, which may allow for milder processing. However, the performance of these low-TI cultivars under reduced heat conditions remains unstudied. This study treated soybean samples under four different temperatures (60, 80, 100, and 121 °C) for 10 min and investigated the impact of heat treatment on TI concentration, in vitro protein digestibility, and nutritional properties of meals from a conventional high-TI variety (Glenn) and a novel low-TI variety (VT Barrack). Results showed that heat treatment at 100 °C significantly improved protein digestibility and lower TI concentrations in both varieties. A negative correlation was observed between protein digestibility and TI concentration in both soybean varieties. At 100 °C, the low-TI variety achieved 81.4% protein digestibility with only 0.6 mg/g TIs, whereas the high-TI variety required 121 °C to achieve comparable protein digestibility and a TI reduction. These findings highlight that low-TI soybeans can lower the necessary thermal treatment to 100 °C to minimize TIs while simultaneously preserving protein quality and cutting energy demand, offering a practical, cost-effective approach to producing higher-quality soybean meals.

## 1. Introduction

Soybean (*Glycine max* (L.) Merrill) is an economically significant protein and oil crop. In 2023, the United States produced 113.3 million metric tons of soybeans, accounting for 28.7% of global production [1], with the global soybean market projected to reach approximately USD 162 billion by 2027, reflecting its growing economic importance [2,3]. Soybeans have significantly higher protein and oil yields compared to other crops, containing 35% protein, 19% oil, 35% carbohydrates (17% of which is dietary fiber), and essential micronutrients (vitamins, minerals, and trace elements) [3,4]. A soybean meal is a co-product of soybean processing after oil extraction, which contains high protein content (44–48%) and a balanced amino acid profile [5,6]. These nutritional properties make a soybean meal a key protein source in animal feed, especially for poultry, swine, and aquaculture. Additionally, it also contributes to the circular economy through applications in biofuels, bioplastics, fertilizers, and functional foods, promoting sustainability and resource efficiency [3].

One major concern for soybean processors is the presence of anti-nutritional factors (ANFs), which significantly inhibit nutrient uptake, thereby lowering the overall nutritional value of soybeans. These ANFs include trypsin inhibitors (TIs), lectins, goitrogens, oligosaccharides, saponins, tannins, phytate, and isoflavones [7,8]. Among them, TIs are the most critical, as they directly interfere with protein digestion by inhibiting the protease activity, leading to pancreatic hyperplasia and metabolic disturbances [9]. TIs are serine protease inhibitors that target enzymes like trypsin, chymotrypsin, elastase, or cathepsin G. They are classified into two protein families: Kunitz trypsin inhibitor (KTI; 18–22 kDa) family and Bowman-Birk trypsin inhibitor (BBTI; 6–9 kDa) family [10]. KTI primarily restrains trypsin activity and has little effect on chymotrypsin. In contrast, BBTI contains two reactive sites capable of inhibiting both trypsin and chymotrypsin [11,12,13,14]. To maximize the nutritional benefits of soybean products, effective strategies for TI inactivation must be developed.

Although various methods, including ultrasound, chemical treatment, and enzymatic degradation, have been explored [15,16,17,18,19,20], thermal treatment is the mainstream treatment for TI inactivation in the soybean processing industry. Thermal energy typically disrupts the physical structure of molecules, making them more susceptible to enzymatic digestion. The thermal inactivation of TIs is influenced by temperature, moisture content, and treatment duration. Traditional heat treatment methods include roasting and boiling, while innovative approaches such as ohmic heating and infrared treatment have also shown effectiveness in reducing TI activity [7]. In industrial processes, soybean meals are steam-treated at a high temperature (110–130 °C) to denature TIs and reduce their activity to a minimal level. However, this process is energy-intensive and may also degrade essential nutrients. An alternative approach is the development of low-TI soybean varieties through breeding, which could reduce the need for high-temperature processing, preserving protein quality while lowering production costs. In 2023, the soybean breeding group at Virginia Tech successfully developed a low-TI line using genome-editing technology. They identified the seed-specific TI genes (KTI1 and KTI3) and knocked them out in the cultivar using CRISPR/Cas9 technology in the soybean. Therefore, the resulting KTI1/KTI3 mutants exhibited a marked reduction in TI content (less than 1 mg/g dry seed) compared to the wild-type (~5 mg/g dry seed). Additionally, the gene knockout did not adversely affect plant growth or maturity under greenhouse conditions [21].

This newly developed soybean line offers us new opportunities for optimizing thermal treatment strategies. However, several critical questions remain unknown: first, to what extent can reduced TI expression lower the required processing temperature of soybean processing? Second, would such a reduction in temperature significantly improve protein digestibility? And third, what is the optimal heat treatment temperature for this low-TI genotype? To address these questions, our study aims to determine the optimal thermal treatment temperature for the low-TI soybean line in order to enhance protein digestibility and reduce energy consumption. In addition, conventional high-TI cultivars were also evaluated to demonstrate the comparative advantage of using low-TI soybean lines in sustainable processing applications.

## 2. Materials and Methods

### 2.1. Plant Material and Chemicals

The soybean varieties *Glenn* (high TI, 6.35 mg total TIs/g soybean seeds) and *VT Barrack* (V12-4590, low TI, 2.24 mg total TIs/g soybean seeds), developed by the Virginia Tech Breeding Program, were used in this study. The following list of chemicals were purchased from Sigma-Aldrich (St. Louis, MO, USA): Trypsin from porcine pancreas (13,000–20,000 BAEE units/mg protein), ɑ-chymotrypsin from bovine pancreas (≥40 units/mg protein), and protease from *Streptomyces griseus* (≥3.5 units/mg protein), hexane, sodium dioxide (NaOH), hydrochloric acid (HCl), β-mercaptoethanol, bovine serum albumin (BSA), copper (II) sulphate pentahydrate, potassium sodium tartrate tetrahydrate, potassium iodide, and sodium hydroxide.

### 2.2. Preparation of Soybean Meals

**Figure 1:**
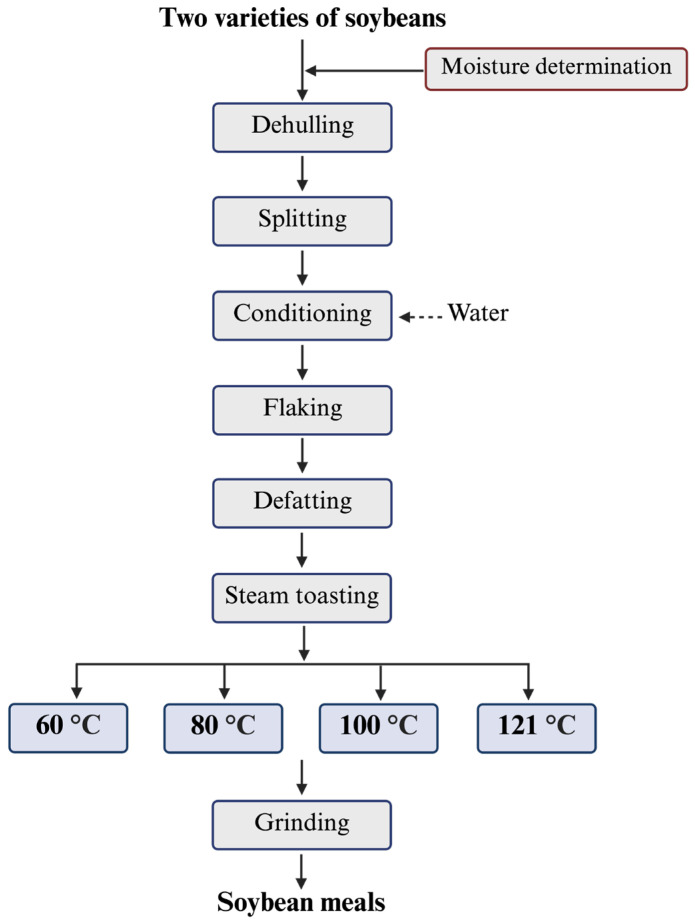
Lab-scale process to produce soybean meal toasted at different temperatures.

Soybean meals were prepared using the developed lab-scale process designed to mimic industrial soybean meal processing, including dehulling and splitting, conditioning, flaking, defatting, toasting, and grinding (Figure 1) [22]. Specifically, 25 g of seeds from each of the two soybean varieties were cracked into 4–5 pieces using a roller mill, followed by hull separation using a dehuller. The moisture content and weight of the samples were measured before transferring them into tubes. Deionized water was added to adjust the moisture content to 15%, and the tubes were placed on a tube rotator (Fisher Scientific, Pittsburgh, PA, USA) at 30 rpm for 10 min, followed by incubation at 65 °C for 15 min. After incubation, the samples were flaked using a roller mill to obtain soybean flakes, then subjected to oil extraction using a Dionex ASE 350 accelerated solvent extractor (Thermo Fisher Scientific, Waltham, MA, USA). The extracted samples were left overnight under a fume hood to allow full hexane evaporation. A custom-modified small-scale steam chamber (25-quart canner cooker) was used to conduct heat treatments. The chamber was designed as a sealed system connected to a high-pressure steam supply (125 PSI) to achieve the target temperatures of 60 °C, 80 °C, 100 °C, and 121 °C. Each variety of soybean meal was steam-treated at these four different temperatures for 10 min. To ensure accurate thermal treatment, a temperature sensor (thermocouple) was inserted into the soybean meal sample and monitored the sample temperature, while a custom-designed steam controller regulated steam input—automatically shutting off the supply upon reaching the set temperature and reopening it when the temperature dropped below the target. In total, ten samples—including eight heat-treated samples (two varieties × four temperatures) and two untreated control samples—were collected. After steaming, the samples were immediately cooled to room temperature and dried at 50 °C to prevent further changes. Finally, the samples were ground into fine powder using an MF 10 basic microfine grinder (IKA Works, Wilmington, NC, USA).

### 2.3. In Vitro Protein Digestibility

Protein digestibility was determined according to the modified method from Hsu et al. [23]. Briefly, a 30 mL soybean meal suspension (containing 6.25 mg soybean meal and 30 mL DI water) and 3 mL of multienzyme solution (comprising 4.8 mg trypsin, 9.3 mg α-chymotrypsin, and 3.9 mg protease dissolving in DI water) were prepared. The pH of both the soybean meal suspension and the multienzyme solution was adjusted to 8.00 (± 0.02) using 1 N HCl or 1 M NaOH. Next, the enzyme solution was mixed with the sample suspension and incubated at 37 °C for 10 min. The pH drop during this time (10 min) was recorded using a pH meter, and protein digestibility was calculated using an equation from Ref. [24]:

(1) $$Protein\;digestibility\%=65.66+18.10\;\times {∆pH}_{10\;min}$$

where ΔpH _10 min_ was the change in pH in 10 min from the initial pH 8.0.

After the enzymatic hydrolysis, the soybean meal suspensions were centrifuged at 8000× *g* for 10 min (model 5804R, Eppendorf, Enfield, CT, USA). The resulting supernatant was collected, freeze-dried, and stored at 4 °C for further analysis.

### 2.4. Quantification of TIs Concentration

The concentrations of TIs in soybean meals were determined using a high-performance liquid chromatography (HPLC) following the procedure developed by Rosso et al. [25]. The grounded soybean powder (10 mg) was mixed with 1.5 mL of 0.1 M sodium acetate buffer (pH 4.5), shaken for 1 h at room temperature, and centrifuged at 12,000× *g* for 15 min. The supernatant (1 mL) was filtered through a syringe with an IC Millex-LG 13 mm mounted 0.2 mm low protein binding hydrophilic polytetrafluoroethylene membrane filter (Millipore, Arklow, Ireland).

KTI was separated using an Agilent 1260 HPLC (Agilent Technologies, Santa Clara, CA, USA) with a POROS R2 guard column (4.6 × 5 mm, 10 μm) and a Poros R2/H analytical column (2.1 × 100 mm, 10 μm). BBTI was separated using the same HPLC system equipped with an Agilent PLRP-S guard column (5 × 3 mm) and analytical column (15 × 4.6 mm, 3 μm). Mobile Phase A was 0.01% (*v*/*v*) trifluoroacetic acid in Milli-Q water, and Phase B was 0.085% (*v*/*v*) trifluoroacetic acid in acetonitrile. Elution was performed at 1 mL/min with the following gradient: 17% B initially, increased to 22% at 3.5 min, 35% at 4 min, 41% at 10 min, 95% at 10.5 min, and returned to 17% at 11 min. The column was maintained at 60 °C, with a 10 μL injection volume, and detection at 220 nm. KTI polymorphic variants had a retention time of 6.6 min.

BBTI was separated on the Agilent HPLC equipped with an Agilent PLRP-S 300 A guard (5 × 3 mm) and column (15 × 4.6 mm, 3 µm). The elution mobile phase A was 0.1% TFA in water, and the B phase was acetonitrile with 0.085% TFA. The gradient elution started at 23% mobile phase B, which increased to 30.2% in 3 min, to 35.2% at 8 min, to 80% at 13 min, and finally returned to 23% at 13.5 min. The column temperature was set at 60 °C, the injection volume was 10 µL, and BBTI isoforms were detected at 220 nm. BBTI isoforms were detected between 5.5 and 7.1 min.

To quantify the concentrations of KTI and BBTI, calibration curves were generated using five concentrations of KTI and BBTI standards (Sigma, St. Louis, MO, USA), ranging from 10 to 150 ppm (µg/mL), specifically, 10, 20, 50, 100, and 150 ppm. These concentrations demonstrated linearity with an R^2^ value of 0.99. The resulting calibration equations were applied to calculate the concentrations of KTI and BBTI in the soybean samples.

### 2.5. Molecular Weight Profile of Soybean Proteins

The molecular weights of original and digested proteins were determined using SDS-PAGE, following the protocol of Hong et al. [26]. with minor modifications. A 50 mg protein sample was mixed with 10 mL PBS buffer (pH 6.8) containing 2% (*w*/*v*) SDS and shaken at 250 rpm for 2 h at room temperature. After centrifugation at 8000× *g* for 5 min, the supernatant was collected. For non-reducing conditions, the protein solution was mixed with 4× Laemmli buffer (Bio-Rad, Hercules, CA, USA) at a 1:3 ratio. For reducing conditions, β-mercaptoethanol was added to the Laemmli buffer at a 1:9 ratio prior to mixing. The mixture was boiled for 5 min and cooled before loading. A 10 μL aliquot was loaded onto a 4–20% Mini-Protean TGX gel (Bio-Rad), with Precision Plus Protein Dual Color Standards (Bio-Rad) as molecular weight markers. Electrophoresis was run at 200 V for 35 min in 1× Tris/Glycine/SDS running buffer. The gel was stained with diluted Brilliant Blue R concentrate (Sigma, St. Louis, MO, USA) for 8 min, rinsed with distilled water overnight, and destained using Bio-Rad’s destaining solution until the background cleared.

The molecular weight of digested protein samples (under non-reducing conditions) was determined using the same method described above, with the enzymes from the digestibility test used as blanks.

### 2.6. Amino Acid Composition Analysis

The amino acid composition was analyzed following the AOAC Official Method 982.30 [27], with results expressed as *w*/*w* % of the sample. For methionine and cystine and/or cysteine determination, the sample (0.1 g) was presoaked overnight in 2 mL cold performic acid at 0–5 °C, followed by the addition of 3 mL cold hydrobromide (HBr) and 0.04 mL of 1-octanol (as a defoaming agent) in an ice bath for 30 min. The mixture was then vacuum-dried at 40 °C for 30 min. After this pretreatment, the dried sample was measured using the same hydrolysis process described above.

For the determination of all amino acids except methionine, cystine, and/or cysteine and tryptophan, a 0.1 g sample was hydrolyzed with 10 mL of 6 M HCl at 110 °C for 24 h. The hydrolysate was then filtered using Whatman No. 1 filter paper (GE Healthcare, Chicago, IL, USA), vacuum dried at 65 °C, and redissolved in sampler buffer for further analysis using an amino acid analyzer.

### 2.7. Protein Solubility

Protein solubility was determined using the Biuret protein assay method [28]. The Biuret reagent was prepared by dissolving 1.5 g copper (II) sulfate pentahydrate and 6 g sodium potassium tartrate in 500 mL of distilled water. Then, 300 mL of 10% (*w*/*v*) NaOH was added, and the volume was adjusted to 1 L with deionized water. Finally, 1 g of potassium iodide was added, and the reagent was stored in the dark. A soybean meal suspension (20 mg/mL) was prepared in deionized water and shaken at 250 rpm for 30 min at room temperature. After centrifugation at 8000× *g* for 10 min, the supernatant was collected. Then, 0.5 mL of the supernatant was mixed with 5 volumes of Biuret reagent, vortexed, and incubated in the dark for 30 min. Absorbance was determined at 540 nm using a UV-VIS scanning spectrophotometer (Shimadzu UV-2101PC, Shimadzu Scientific Instruments, Columbia, MD, USA).

Protein concentrations were quantified using a calibration curve generated with BSA standards (0 to 20 mg/mL), prepared by diluting 20 mg/mL of BSA solution with deionized water. The calibration equation (R^2^ ≈ 0.99) was used to calculate protein concentrations, and the dissolved protein weight was calculated based on the supernatant volume. Soybean meal solubility was then calculated using the following equation:

(2) $$Protein\;solubility\%=\;\frac{Protein\;weight\;in\;supernatant}{Protein\;weight\;in\;original\;sample}\;\times 100$$

### 2.8. Color Test

The color of soybean meal samples was measured using a Minolta Chroma Meter CR-300 colorimeter (Minolta Camera Co., Osaka, Japan). The L* (lightness), a* (redness to greenness), and b* (yellowness to blueness) values were collected and averaged from three replicates.

### 2.9. Statistical Analysis

All experiments except for amino acid composition analysis and molecular weight analysis were conducted in triplicate, and all data were presented as mean values, and variability was illustrated using error bars in the figures. The error bars represent the standard deviation (SD) of at least three independent replicates. Statistical analysis was performed using IBM SPSS Statistics software (version 27.0.1., Armonk, NY, USA). One-way analysis of variance (ANOVA) was used to assess differences, and multiple comparisons were conducted using Tukey’s test. Significant differences were considered when *p <* 0.05. A Pearson correlation analysis was performed to determine the relationships between KTI concentration and digestibility, BBTI concentration and digestibility, and total TI and digestibility in IBM SPSS Statistics software (version 27.0.1., Armonk, NY, USA).

## 3. Results and Discussion

### 3.1. In Vitro Protein Digestibility, TI Concentrations, and Correlation Analysis

**Figure 2:**
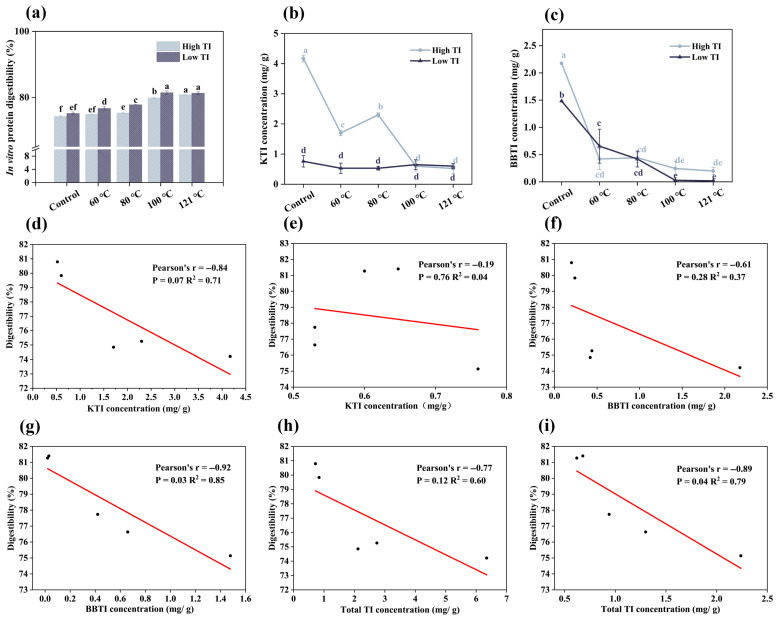
In vitro protein digestibility of protein in soybean meals toasted at temperature from 60 to 121 °C (**a**). Concentration of TIs of protein in soybean meals toasted at temperature from 60 to 121 °C (**b**,**c**). Correlation analysis of high-TI variety between digestibility and KTI, BBTI, and total TIs concentration (**d**,**f**,**h**). Correlation analysis of low-TI variety between digestibility and KTI, BBTI, and total TIs concentration (**e**,**g**,**i**). Different letters indicate significant differences in the same column (n = 3, *p <* 0.05). TI: trypsin inhibitor, High TI: soybean meal samples with high-TI concentration, low TI: soybean meal samples with low-TI concentration, KTI: Kunitz trypsin inhibitor, BBTI: Bowman–Birk trypsin inhibitor.

Protein digestibility reflects the efficiency of gastrointestinal proteases in hydrolyzing proteins into absorbable amino acids and peptides. It is a key factor in assessing soybeans’ nutritional quality, as poor digestibility may contribute to malnutrition [29]. The results showed that the protein digestibility of the untreated soybean meals was 74.21% and 75.13% for high-TI and low-TI varieties, respectively (Figure 2a). However, heat treatment significantly improved protein digestibility (*p <* 0.05) (Figure 2a). For the high-TI variety, protein digestibility increased consistently from 74.85% to 80.79% as the treatment temperature increased from 60 °C to 121 °C. In the low-TI variety, protein digestibility increased from 76.63% to 81.4% as the temperature increased from 60 °C to 100 °C, but further heating to 121 °C did not enhance protein digestibility, indicating that 100 °C is sufficient for optimizing protein digestibility for the low-TI variety. Notably, the digestibility of the low-TI variety at 100 °C exceeded that of the high-TI variety at 121 °C, supporting our hypothesis that the low-TI variety would achieve superior protein digestibility with a lower energy input. The heat treatment partially unfolds protein structures, exposing active sites and enhancing protease accessibility, thereby improving digestibility [30,31]. Additionally, the high temperatures will also cause the degradation of the TIs, reducing their inhibition of digestive enzymes [32]. A similar research by Tang et al. [33] used soybean protein as an alternative to infant formula and investigated the effect of heat treatment on the digestibility of soy protein, and found a significant increase in digestibility from 30% to 60% at 100 °C. Overall, we can conclude that lower heat treatment is required to optimize soybean meal digestibility for the low-TI soybean variety compared to the high-TI variety, potentially reducing energy consumption during processing. However, the exact reduction range should be further investigated in future studies.

Since TIs in soybean meals play a key role in protein digestibility, we measured the concentrations of KTI and BBTI in soybean meals (Figure 2b,c). KTI concentration in the high-TI variety decreased significantly from 4.17 (control: untreated) to 0.60 mg/g at 100 °C (*p <* 0.05) and remained stable (0.52 mg/g) when the temperature continued to rise to 121 °C (Figure 2b). In contrast, the KTI concentration of the low-TI variety samples was relatively stable at a low level, remaining around 0.65 mg/g. The BBTI concentration in high-TI variety decreased significantly at 60 °C, from 2.18 mg/g to 0.42 mg/g (*p <* 0.05), and stabilized at higher temperatures, reaching 0.20 mg/g at 121 °C (Figure 2c). Conversely, the low-TI variety showed a continuous decrease from 1.48 mg/g (control) to 0.03 mg/g at 100 °C (*p <* 0.05), stabilizing after 100 °C (reached 0.02 mg/g at 121 °C). Notably, after 80 °C, the BBTI concentration in the low-TI variety was lower than that in the high-TI variety, which indicates that temperatures above 80 °C were more suitable for reducing the BBTI content in the low-TI variety. Compared to KTI, BBTI exhibited less reduction, which may be due to its rigid structures, which led to greater thermal stability [34].

Correlation analysis examined the relationship between KTI, BBTI, and total TIs concentrations (sum of KTI and BBTI) and protein digestibility (Figure 2d–i). For the high-TI variety (Figure 2d,f,h), moderate negative correlations were observed between KTI (r = −0.84, R^2^ = 0.71), BBTI (r = −0.61, R^2^ = 0.37), total TI concentrations (r = −0.77, R^2^ = 0.60), and protein digestibility, indicating both KTI and BBTI concentrations appeared to influence protein digestibility. In contrast, for the low-TI variety (Figure 2e,g,i), the correlations were also negative but weaker, with r values of −0.19 (KTI), −0.92 (BBTI), and −0.89 (total TIs), and corresponding R^2^ values of 0.04, 0.85, and 0.79, respectively. The weak correlation between the KTI concentration and the protein digestibility of the low-TI variety indicated that KTI content had little influence on the protein digestibility in low-TI samples. This can be due to the fact that the KTI content in the low-TI variety is extremely low even before the heat treatment; meanwhile, the BBTI content in the low-TI variety is high; thus, the protein digestibility is governed by the BBTI content rather than the KTI content. Overall, the results indicate that TI concentrations are inversely associated with protein digestibility in both soybean varieties, but the strength of this relationship depends on the initial TI content in each variety.

Mechanistically, during digestion, trypsin is activated to break down proteins, but the presence of TIs leads to the formation of stable complexes with trypsin by several weak bonds. This binding reduces the availability of free trypsin in the intestine, interfering with normal protein digestion [14,35]. Consistent with these findings, previous studies have reported moderate or strong negative correlations between TIs concentration and digestibility, with correlation coefficient values ranging from −0.41 to −0.82 [34]. Notably, while the r values indicate moderate negative relationships, it is important to note that the R^2^ values (varied from 0.04 to 0.85) suggest that the majority of the variation in protein digestibility remains unexplained by TI concentrations alone. This implies that other factors, such as heat-induced protein denaturation, or reactions between other components, may also play substantial roles in determining digestibility.

### 3.2. Molecular Weight Profile of Soybean Meal Proteins

**Figure 3:**
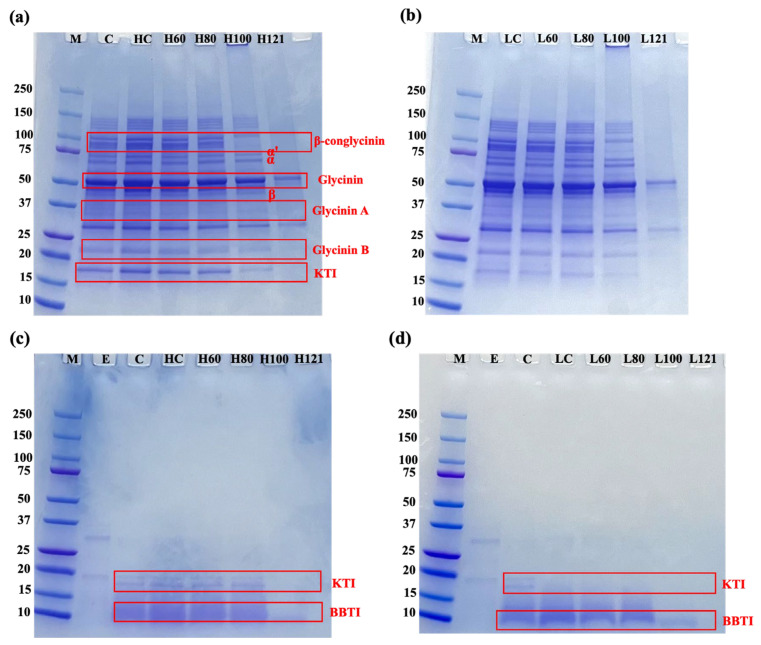
The non-reducing SDS-PAGE patterns of original soybean samples and digested soybean samples toasted at temperature from 60 to 121 °C. (**a**,**b**) shows original soybean samples and (**c**,**d**) shows digested soybean samples. M: molecular weight standard, E: enzymes control, C: commercial soybean sample, TI: trypsin inhibitor, HC: high-TI control, LC: low-TI control, H: soybean meal samples with high-TI concentration, L: soybean meal samples with low-TI concentration, KTI: Kunitz trypsin inhibitor, BBTI: Bowman–Birk trypsin inhibitor.

The SDS-PAGE analysis is an effective method to evaluate the molecular weight distribution of proteins in soybean meals. The SDS-PAGE profiles under both non-reducing and reducing conditions are shown in Figure 3a,b and Figure S1. The protein profiles of the two soybean varieties (low-TI and high-TI) were similar and aligned with previous findings [33,36,37]. The primary soybean protein fractions, their subunits, and TIs were clearly identifiable in the profiles (Figure 3a,b). Soybean proteins are primarily categorized into two major fractions: β-conglycinin (7S) and glycinin (11S). β-Conglycinin consists of three subunits (α, α′, and β), while glycinin contains subunits composed of acidic and basic polypeptides connected by disulfide bonds, named glycinin A and B, respectively [38,39]. The KTI bands were observed at approximately 20 kDa in non-reducing gels, consistent with findings previously reported [19], while BBTI bands were only detected around 10 kDa under reducing conditions. This difference may be attributed to the more rigid structure of BBTI, requiring reducing agents for degradation.

Under reducing conditions (Figure S1), the band intensities were generally higher compared to non-reducing conditions, with concentrated bands observed around 60–100 kDa, 25–37 kDa, and 15–20 kDa. In contrast, under non-reducing conditions, major bands were observed in the 75–100 kDa and around 50 kDa regions. Fewer bands were observed in the 100 to 150 kDa and around 50 kDa range under non-reducing conditions, indicating that some cross-linked proteins were degraded by the reducing agent and separated into smaller polymers. Additionally, partial disulfide bond cleavage in glycinin subunits led to the formation of acidic or basic peptides.

Heat treatment at 100 °C and 121 °C caused significant changes in all the protein profiles, resulting in notably lower band intensities and fewer bands compared to those treated at lower temperatures (in the provided temperature range) (Figure 3a,b). Notably, the intensity of KTI bands significantly decreased with increasing temperature, reinforcing the findings on protein digestibility and TI reduction. Additionally, some soluble aggregates were observed at the top of the non-reducing gels in samples treated at 100 °C (Figure 3a,b, H100, and L100 lane), likely due to their large size preventing gel entry. However, these aggregates were absent under reducing conditions (Figure S1), indicating their formation was caused by disulfide bond cross-linking, consistent with previous reports [33]. At 121 °C, most bands disappeared, indicating extensive aggregation and reduced solubility due to excessive heating. This observation aligns with the solubility results discussed later. Similarly, Zhang et al. [40] reported that heating soy milk at 95–100 °C for 10 min led to soluble aggregate formation with a hydrophobic core and surrounding α, α′, and acidic polypeptides. However, at higher dry-heating temperatures (160 °C and 200 °C), insoluble aggregates formed via non-covalent and covalent interactions, respectively. These findings suggest that excessive heat-induced aggregation negatively affects protein digestibility and quality, emphasizing the importance of optimizing thermal treatments to maintain protein functionality.

SDS-PAGE profiles of digested soybean meals under non-reducing conditions were also analyzed (Figure 3c,d). Compared to untreated soybean samples, digested samples exhibited lower molecular weights and fewer bands, though faint KTI and BBTI bands were still visible, indicating that some TIs remained intact and could still affect the protein digestibility. Notably, the high-TI variety showed more distinct KTI and BBTI bands compared with the low-TI variety, highlighting the inherent differences in TI content between the two. However, samples treated at 100 °C and 121 °C showed the fewest bands, in some cases, no detectable bands, suggesting that KTI and BBTI were either degraded at higher temperatures or hydrolyzed into peptides too small to be detected within the 10–250 KDa molecular weight range.

### 3.3. Amino Acid Composition

Plant proteins serve as a rich source of amino acids, which play a crucial role in food and nutrition. The amino acid composition of the two soybean meal varieties treated at 80 °C, 100 °C, and 121 °C was analyzed (Table 1). Overall, amino acid levels exhibited only minor variations. Lysine, an essential amino acid critical for human nutrition, exhibited only minor variations in both varieties. Methionine, a sulfur-containing and limiting amino acid in soybeans, constrains the nutritional value of soy protein in animal feeds. Therefore, supplementation with methionine is often recommended to balance diets for both animal and human nutrition [41]. The non-essential amino acids were the most abundant in the amino acid composition profile and remained largely unaffected by increasing temperatures. Meanwhile, the non-proteinogenic amino acids were either present in low concentrations or undetectable in all samples. Overall, the amino acid composition had limited changes, indicating that high temperatures do not break down the primary structure of soy proteins. Similar results were reported by Nasri et al. [42], who found that roasting and steeping had minimal effects on the total amino acid content and composition of soybeans, and no systematic effects were observed between varieties. It should be noted, however, that amino acid measurements in this study were based on a single replicate, which limits the statistical confidence of the conclusions. Future studies should include additional replicates to improve the reliability and robustness of the findings.

### 3.4. Protein Solubility

**Figure 4:**
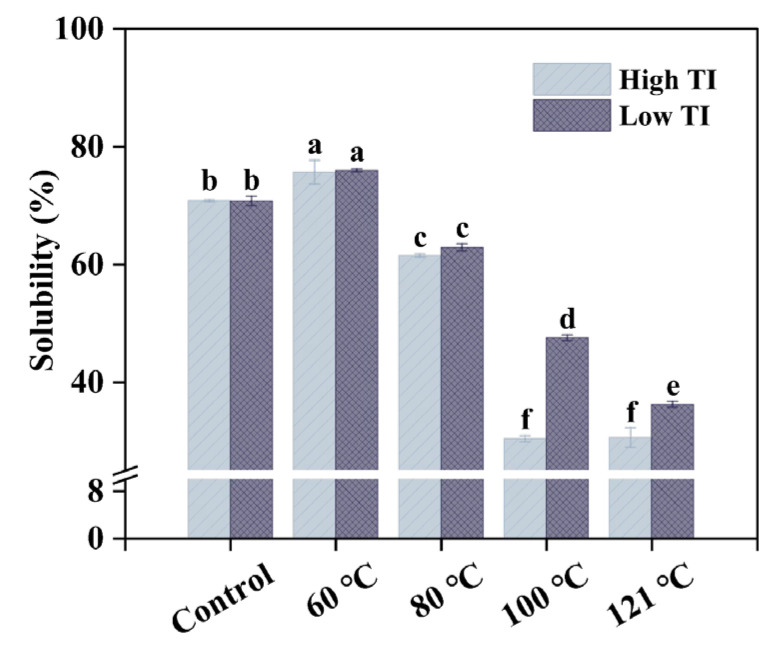
Protein solubility in soybean meals toasted at temperature from 60 to 121 °C. Different letters indicate significant differences in the same column (n = 3, *p <* 0.05). TI: trypsin inhibitor, high TI: soybean meal samples with high-TI concentration, low TI: soybean meal samples with low-TI concentration.

Protein solubility is essential for the physicochemical properties, sensory attributes, shelf life, and nutritional quality of protein-rich foods. It also plays a key role in food production, affecting phase stability and functional properties such as emulsification and foaming by influencing protein migration to oil-water or air-water interfaces [43]. The solubility of soybean meal samples at the as-is pH was analyzed and is shown in Figure 4.

Both high- and low-TI soybean varieties exhibited a similar solubility trend with increasing temperature. Solubility significantly increased at 60 °C (*p <* 0.05), rising from 70.8% to 75.7% in the high-TI variety and from 70.8% to 75.9% in the low-TI variety. This enhancement could be attributed to the partial protein unfolding, which exposes hydrophilic regions, enhancing the interaction with water. However, as temperatures increased further, solubility declined dramatically (*p <* 0.05), with values dropping to 30.6% (high TI) and 36.2% (low TI). These findings are consistent with previous studies by Zhang et al. [44], who reported that higher temperatures reduce soybean protein solubility due to protein aggregation via non-covalent interactions. Similarly, Žilić et al. [45] reported that soybean protein solubility decreases with thermal processing. One possible explanation is that heat-induced protein unfolding increased the exposure of hydrophobic areas on the protein surface, leading to decreased solubility [29]. However, this speculation is contradicted by the results observed at 60 °C, indicating that the balance between exposed hydrophilic and hydrophobic regions is highly dependent on the degree of unfolding and specific conditions. In addition, it is interesting to note that the solubilities of protein in the low-TI variety are significantly higher than those in the high-TI variety at 100 °C-treatment, which may have contributed to the higher protein digestibility of the low-TI variety, as shown in Figure 2a. Moreover, we also found the influence of solubility on protein digestibility (r = −0.87). However, digestibility may be affected by multiple factors, such as protein conformation or TI levels. Further studies are needed to clarify the underlying mechanisms.

### 3.5. Color Test

**Figure 5:**
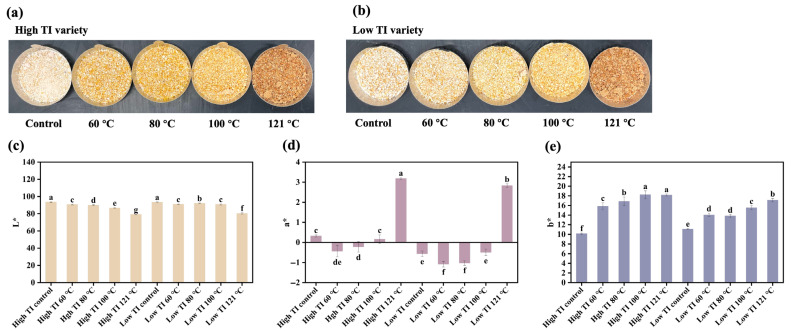
Color of soybean meals toasted at temperature from 60 to 121 °C. Different letters indicate significant differences in the same column (n = 3, *p* < 0.05). TI: trypsin inhibitor, high TI: soybean meal samples with high-TI concentration, low TI: soybean meal samples with low-TI concentration.

Color is a key quality parameter influenced by processing conditions, reflecting chemical and physical changes in proteins, sugars, and other components. The color measurements of soybean meal samples are shown in Figure 5, with L*, a*, and b* values describing lightness (0–100), red to green color (negative to positive), and yellow to blue color (negative to positive), respectively. The results demonstrated that processing temperature significantly affected the color of soybean meal in both varieties. The L* value decreased significantly with increasing temperature (*p <* 0.05), dropping from 93.6 to 79.2 in the high-TI variety and from 93.2 to 80.4 in the low-TI variety (Figure 5c). This darkening effect at 121 °C is likely due to the formation of pigments through caramelization [46]. Similar observations were reported by Dahmer et al. [47], who observed a significant decrease in soybean lightness after heat treatment. Likewise, Zhang et al. [40] also showed a dark brown coloration in soybean samples heated at 200 °C, further supporting the occurrence of the Millard reaction. The a* value initially decreased at 60 °C and 80 °C, indicating a more reddish hue, but increased significantly at 121 °C (*p <* 0.05), shifting toward a more greenish color (Figure 5d). Meanwhile, the b* values increased with temperature in both varieties, ranging from 10.16 to 18.16 for high-TI variety samples and from 11.1 to 17.1 for low-TI variety samples (Figure 5e). This shift suggests that as temperature rises, soybean meals take on a more bluish tone.

## 4. Conclusions

In summary, this study investigated the effects of heat treatments on protein digestibility, TIs concentration, protein solubility, amino acid composition, and color in high- and low-TI soybean varieties. Results showed that the low-TI variety (VT Barrack) achieved high digestibility at 100 °C with minimal TI levels, while the high-TI variety (Glenn) required 121 °C to reach comparable outcomes. Correlation analysis revealed moderate negative relationships between TI concentration and digestibility in the high-TI variety, while the low-TI variety exhibited a slightly weaker trend. These findings highlighted the importance of reducing these inhibitors to improve nutritional quality. Protein solubility increased with heat treatment, while the amino acid profile remained stable across treatments, indicating that high temperatures did not degrade the primary protein structure. In contrast, darker color development at higher temperatures suggests potential quality losses.

These findings support the hypothesis that cultivars with low-TI expression enable effective TI inactivation under milder processing conditions. However, there are still some limitations. As a genetically modified (GM) soybean line, its commercial cultivation is subject to region-specific regulatory approvals. While countries like the United States, Brazil, and Argentina have authorized the commercial cultivation of multiple GM soybean varieties [48], the European Union currently does not permit the cultivation of GM soybean; only several events are approved for import and use in food and feed [49]. These regulatory differences may limit the real-world applicability of this line in some markets.

Overall, this study effectively demonstrates a promising cost- and energy-efficient approach for downstream processing by reducing TI content at the breeding stage.

## 5. Future Perspectives

While this study focused on specific soybean varieties and processing conditions, further research should explore a broader range of varieties and processing methods and analyze functional properties, sensory attributes, and storage stability. Incorporating in vivo digestibility tests and advanced proteomic techniques such as LC-MS/MS would offer deeper insights into the bioavailability of heat-treated soy proteins and identify digestion-resistant peptides. These approaches could strengthen the understanding of protein degradation mechanisms and support more precise processing optimization.

## Figures and Tables

**Table 1:** Amino acid composition of soybean meals processed at toasting temperatures from 80 to 121 °C. Note: TI: trypsin inhibitor, high TI: soybean meal samples with high-TI concentration, low TI: soybean meal samples with low-TI concentration.

Amino Acids (*w*/*w*%)	High TI 80 °C	High TI 100 °C	High TI 121 °C	Low TI 80 °C	Low TI 100 °C	Low TI 121 °C
**Essential amino acids**						
Histidine	1.18	1.28	1.13	1.21	1.26	1.26
Isoleucine	2.26	2.28	2.27	2.18	2.32	2.33
Leucine	3.44	3.55	3.42	3.36	3.55	3.64
Lysine	2.73	3.00	2.94	2.88	3.00	2.85
Methionine	0.62	0.64	0.64	0.60	0.66	0.65
Phenylalanine	2.27	2.36	2.27	2.23	2.38	2.41
Threonine	1.74	1.78	1.70	1.66	1.75	1.79
Tryptophan	0.45	0.33	0.47	0.48	0.35	0.51
Valine	2.31	2.33	2.33	2.25	2.40	2.42
**Non-essential amino acids**						
Alanine	1.95	2.04	1.93	1.90	2.04	2.06
Arginine	3.03	3.24	3.22	3.06	3.39	3.33
Aspartic Acid	5.15	5.27	5.24	5.01	5.33	5.37
Cysteine	0.59	0.67	0.66	0.59	0.68	0.60
Glutamic Acid	8.25	8.68	8.54	8.31	8.62	8.95
Glycine	1.90	1.91	1.90	1.84	1.95	1.99
Proline	2.21	2.29	2.21	2.19	2.29	2.34
Serine	1.83	1.90	1.90	1.76	1.85	1.90
Tyrosine	1.28	1.40	1.24	1.22	1.75	1.76
**Non-proteinogenic amino acids**						
Taurine	0.08	0.09	0.08	0.10	0.12	0.13
Hydroxyproline	0.00	0.00	0.00	0.00	0.00	0.00
Lanthionine	0.00	0.00	0.00	0.00	0.00	0.00
Ornithine	0.06	0.06	0.03	0.05	0.05	0.09
Hydroxylysine	0.00	0.06	0.02	0.03	0.06	0.02
Total	42.91	45.80	46.40	43.33	45.16	44.14

## Data Availability

The original contributions presented in this study are included in the article. Further inquiries can be directed to the corresponding author.

## Supplementary Materials

The following supporting information can be downloaded at https://www.mdpi.com/article/10.3390/foods14173039/s1, Figure S1: The reducing SDS-PAGE patterns of original soybean samples toasted at temperature from 60 to 121 °C. Note: (a) shows high-TI soybean samples and (b) shows low-TI soybean samples. M: molecular weight standard, C: commercial soybean sample, TI: trypsin inhibitor, HC: high-TI control, LC: low-TI control, H: soybean meal samples with high-TI concentration, L: soybean meal samples with low-TI concentration, KTI: Kunitz trypsin inhibitor, BBTI: Bowman–Birk trypsin inhibitor.

## Abbreviations

The following abbreviations are used in this manuscript:

ANFs	Anti-nutritional factors
TI	Trypsin inhibitor
BBTI	Bowman–Birk trypsin inhibitor
KTI	Kunitz trypsin inhibitor
SDS	Sodium dodecyl sulfate
PAGE	Polyacrylamide gel electrophoresis
