# Supplementary material for: Reducing Heat Without Impacting Quality: Optimizing Trypsin Inhibitor Inactivation Process in Low-TI Soybean
Source: Foods. 2025 Aug 29;14(17):3039. doi: 10.3390/foods14173039 (PMC12428520; doi:10.3390/foods14173039)
Supplement: Supplementary file 1 [file foods-14-03039-s001.zip › foods-3702558-supplementary.pdf]

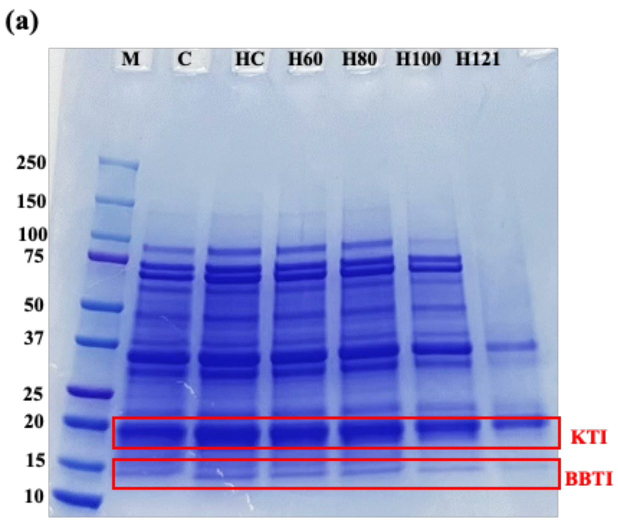

Figure S1. The reducing SDS-PAGE patterns of original soybean samples toasted at temperature from 60 to 121 °C. Note: (a) shows high TI soybean samples and (b) shows low TI soybean samples. M: molecular weight standard, C: commercial soybean sample, TI: trypsin inhibitor, HC: high TI control, LC: low TI control, H: soybean meal samples with high TI concentration, L: soybean meal samples with low TI concentration, KTI: Kunitz trypsin inhibitor, BBTI: Bowman-Birk trypsin inhibitor.
